# Chlorido­tetra­kis­(imidazole)­copper(II) chloride. Corrigendum

**DOI:** 10.1107/S2056989021002267

**Published:** 2021-03-31

**Authors:** Ting Bin Li, Ya Li Hu, Ji Kun Li, Guo Fang He

**Affiliations:** a Department of Materials Science and Technology, Taishan University, Taian 271021, People’s Republic of China

## Abstract

In the article by Li *et al.* [*Acta Cryst.* (2007), E**63**, m2536], four imidazole H atoms are missing in the refinement.<!?tpb=20pt>

## Abstract 2

Corrigendum to *Acta Cryst.* (2007), E**63**, m2536.

The structure of chlorido­tetra­kis­(imidazole)­copper(II) chlo­ride, reported in the article by Li *et al.* (2007 ▸), has been rerefined to include four missing imidazole H atoms. The crystal was twinned by pseudomerohedry, which was dealt with using standard *SHELXL* methods (TWIN and BASF commands). The revised crystal data, data collection and structure refinement details are summarized in Table 1 ▸ and the revised chemical drawing is shown in Fig. 1 ▸.<!?tpb=-40pt>

## Supplementary Material

Crystal structure: contains datablock(s) I, global. DOI: 10.1107/S2056989021002267/me6126sup1.cif

Structure factors: contains datablock(s) I. DOI: 10.1107/S2056989021002267/me6126Isup2.hkl

CCDC reference: 2066475

Additional supporting information:  crystallographic information; 3D view; checkCIF report

## Figures and Tables

**Figure 1:**
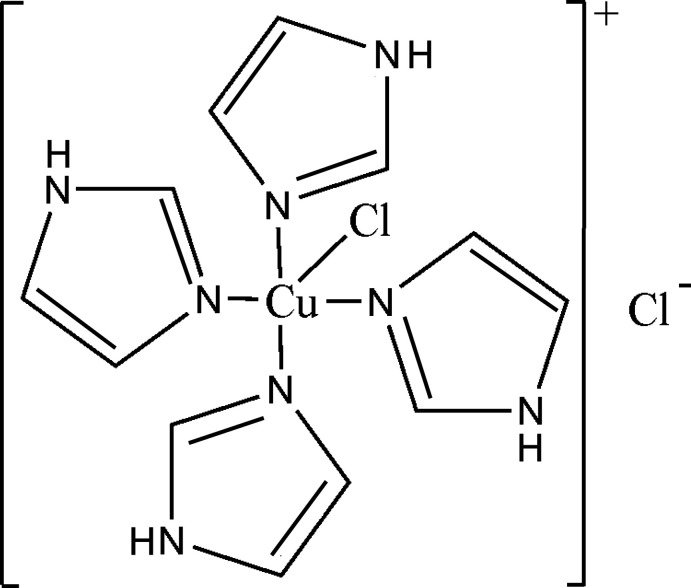
Chemical scheme for chlorido­tetra­kis­(imidazole)­copper(II) chlo­ride.

**Table 1:** Experimental details

Crystal data	
Chemical formula	[CuCl(C_3_H_4_N_2_)_4_]Cl
*M*_r_	406.77
Crystal system, space group	Monoclinic, *P*2_1_/*n*
Temperature (K)	293
*a*, *b*, *c* (Å)	8.8662 (3), 13.3199 (4), 13.9190 (4)
β (°)	90.042 (1)
*V* (Å^3^)	1643.79 (9)
*Z*	4
Radiation type	Mo *K*α
μ (mm^−1^)	1.67
Crystal size (mm)	0.15 × 0.12 × 0.10

Data collection	
Diffractometer	Bruker CCD
Absorption correction	Multi-scan (*SADABS*; Krause *et al.*, 2015 ▸)
*T*_min_, *T*_max_	0.788, 0.851
No. of measured, independent and observed [*I* > 2σ(*I*)] reflections	18819, 3317, 2798
*R*_int_	0.039
(sin θ/λ)_max_ (Å^−1^)	0.650

Refinement	
*R*[*F* ^2^ > 2σ(*F* ^2^)], *wR*(*F* ^2^), *S*	0.026, 0.057, 0.94
No. of reflections	3317
No. of parameters	209
H-atom treatment	H-atom parameters constrained
Δρ_max_, Δρ_min_ (e Å^−3^)	0.25, −0.37

Computer programs: *SMART* (Siemens, 1996 ▸), *SAINT* (Siemens, 1996 ▸), *SHELXT* (Sheldrick, 2015*a* ▸) and *SHELXL2018* (Sheldrick, 2015*b* ▸).

## supplementary crystallographic information

### Crystal data

**Table:**

[CuCl(C_3_H_4_N_2_)_4_]Cl	*F*(000) = 828
*M**_r_* = 406.77	*D*_x_ = 1.644 Mg m^−^^3^
Monoclinic, *P*2_1_/*n*	Mo *K*α radiation, λ = 0.71073 Å
*a* = 8.8662 (3) Å	Cell parameters from 8636 reflections
*b* = 13.3199 (4) Å	θ = 2.7–27.4°
*c* = 13.9190 (4) Å	µ = 1.67 mm^−^^1^
β = 90.042 (1)°	*T* = 293 K
*V* = 1643.79 (9) Å^3^	Block, blue
*Z* = 4	0.15 × 0.12 × 0.10 mm

### Data collection

**Table:**

Bruker CCD diffractometer	3317 independent reflections
Radiation source: fine-focus sealed-tube	2798 reflections with *I* > 2σ(*I*)
Detector resolution: 9.1 pixels mm^-1^	*R*_int_ = 0.039
φ and ω scans at fixed χ = 55°	θ_max_ = 27.5°, θ_min_ = 1.5°
Absorption correction: multi-scan (*SADABS*; Krause *et al.*, 2015)	*h* = −11→11
*T*_min_ = 0.788, *T*_max_ = 0.851	*k* = −17→17
18819 measured reflections	*l* = −17→17

### Refinement

**Table:**

Refinement on *F*^2^	Primary atom site location: structure-invariant direct methods
Least-squares matrix: full	Secondary atom site location: difference Fourier map
*R*[*F*^2^ > 2σ(*F*^2^)] = 0.026	Hydrogen site location: difference Fourier map
*wR*(*F*^2^) = 0.057	H-atom parameters constrained
*S* = 0.94	*w* = 1/[σ^2^(*F*_o_^2^) + (0.022*P*)^2^] where *P* = (*F*_o_^2^ + 2*F*_c_^2^)/3
3317 reflections	(Δ/σ)_max_ = 0.001
209 parameters	Δρ_max_ = 0.25 e Å^−^^3^
0 restraints	Δρ_min_ = −0.37 e Å^−^^3^

### Special details

**Table:**

Geometry. All esds (except the esd in the dihedral angle between two l.s. planes) are estimated using the full covariance matrix. The cell esds are taken into account individually in the estimation of esds in distances, angles and torsion angles; correlations between esds in cell parameters are only used when they are defined by crystal symmetry. An approximate (isotropic) treatment of cell esds is used for estimating esds involving l.s. planes.
Refinement. Refined as a two-component twin.

### Fractional atomic coordinates and isotropic or equivalent isotropic displacement parameters (Å^2^)

**Table:**

	*x*	*y*	*z*	*U*_iso_*/*U*_eq_	
Cu1	0.68666 (3)	0.28667 (2)	0.37745 (2)	0.02393 (7)	
N1	0.6959 (2)	0.28765 (13)	0.23419 (13)	0.0259 (4)	
N2	0.6679 (2)	0.32558 (15)	0.08280 (14)	0.0348 (5)	
H2N	0.636864	0.355523	0.031725	0.042*	
N3	0.58536 (18)	0.15168 (11)	0.37424 (14)	0.0261 (4)	
N4	0.4040 (2)	0.04018 (13)	0.37092 (16)	0.0356 (4)	
H4N	0.314625	0.015090	0.369077	0.043*	
N5	0.6952 (2)	0.27930 (12)	0.52089 (13)	0.0279 (4)	
N6	0.6741 (3)	0.31834 (16)	0.67249 (14)	0.0378 (5)	
H6N	0.649042	0.350481	0.723635	0.045*	
N7	0.85774 (18)	0.38627 (12)	0.37990 (14)	0.0272 (4)	
N8	1.0848 (2)	0.44900 (14)	0.37450 (16)	0.0391 (5)	
H8N	1.181520	0.452439	0.371463	0.047*	
C1	0.6293 (3)	0.34862 (18)	0.17279 (17)	0.0307 (5)	
H1	0.564507	0.400524	0.189836	0.037*	
C2	0.7649 (3)	0.24628 (19)	0.08597 (19)	0.0388 (6)	
H2	0.810447	0.214439	0.034083	0.047*	
C3	0.7813 (3)	0.22351 (17)	0.17973 (17)	0.0332 (6)	
H3	0.841423	0.172116	0.203825	0.040*	
C4	0.4387 (2)	0.13815 (16)	0.37156 (17)	0.0306 (5)	
H4	0.368157	0.189789	0.370286	0.037*	
C5	0.5354 (2)	−0.01236 (16)	0.3737 (2)	0.0391 (5)	
H5	0.546237	−0.081805	0.374264	0.047*	
C6	0.6467 (2)	0.05655 (15)	0.3755 (2)	0.0359 (5)	
H6	0.749319	0.042122	0.377214	0.043*	
C7	0.6409 (3)	0.34477 (18)	0.58258 (18)	0.0334 (6)	
H7	0.586607	0.401851	0.565718	0.040*	
C8	0.7550 (3)	0.2312 (2)	0.6690 (2)	0.0421 (7)	
H8	0.793436	0.195461	0.720941	0.050*	
C9	0.7680 (3)	0.20719 (18)	0.57536 (18)	0.0354 (6)	
H9	0.817889	0.151193	0.551229	0.042*	
C10	1.0020 (2)	0.36497 (17)	0.3752 (2)	0.0347 (5)	
H10	1.041431	0.300308	0.372764	0.042*	
C11	0.9878 (3)	0.52801 (17)	0.3794 (2)	0.0415 (6)	
H11	1.012833	0.595805	0.380273	0.050*	
C12	0.8483 (2)	0.48821 (16)	0.3828 (2)	0.0367 (5)	
H12	0.759181	0.524784	0.386633	0.044*	
Cl1	0.44359 (6)	0.39845 (4)	0.37917 (5)	0.03331 (12)	
Cl2	1.05460 (6)	0.09206 (4)	0.37508 (5)	0.03696 (13)	

### Atomic displacement parameters (Å^2^)

**Table:**

	*U*^11^	*U*^22^	*U*^33^	*U*^12^	*U*^13^	*U*^23^
Cu1	0.02695 (13)	0.02467 (12)	0.02018 (13)	−0.00402 (10)	−0.00038 (13)	−0.00037 (12)
N1	0.0273 (10)	0.0266 (9)	0.0237 (10)	−0.0037 (8)	−0.0006 (8)	0.0001 (7)
N2	0.0360 (11)	0.0460 (12)	0.0225 (10)	−0.0039 (10)	−0.0034 (9)	0.0096 (9)
N3	0.0288 (9)	0.0259 (8)	0.0236 (9)	−0.0016 (7)	0.0004 (9)	−0.0013 (9)
N4	0.0293 (9)	0.0336 (10)	0.0440 (12)	−0.0093 (8)	−0.0001 (10)	−0.0009 (10)
N5	0.031 (1)	0.0302 (9)	0.0225 (10)	−0.0027 (8)	0.0000 (9)	−0.0005 (8)
N6	0.0375 (12)	0.0557 (13)	0.0202 (10)	−0.0025 (11)	0.0028 (10)	−0.0095 (9)
N7	0.0291 (9)	0.0289 (9)	0.0236 (9)	−0.0028 (7)	−0.0004 (9)	0.0008 (8)
N8	0.0235 (9)	0.0516 (11)	0.0421 (11)	−0.0108 (8)	0.0014 (11)	−0.0032 (12)
C1	0.0297 (13)	0.0338 (13)	0.0286 (13)	−0.0002 (10)	−0.0006 (10)	0.0024 (10)
C2	0.0491 (17)	0.0373 (13)	0.0301 (14)	−0.0023 (11)	0.0079 (12)	−0.0052 (11)
C3	0.0391 (15)	0.0295 (12)	0.0310 (14)	0.0038 (10)	0.0036 (11)	0.0003 (10)
C4	0.0313 (11)	0.0302 (11)	0.0303 (12)	0.0012 (9)	−0.0010 (12)	0.0011 (10)
C5	0.0376 (12)	0.0234 (10)	0.0563 (15)	−0.0005 (9)	−0.0038 (14)	0.0010 (13)
C6	0.0296 (11)	0.0304 (10)	0.0477 (14)	0.0029 (8)	0.0006 (13)	−0.0008 (13)
C7	0.0325 (14)	0.0356 (13)	0.0321 (13)	−0.0019 (10)	−0.0009 (10)	−0.0044 (10)
C8	0.0417 (15)	0.0546 (17)	0.0299 (15)	0.0018 (13)	−0.0061 (12)	0.0089 (12)
C9	0.0389 (14)	0.0367 (13)	0.0306 (14)	0.0048 (11)	−0.0027 (11)	0.0012 (10)
C10	0.0337 (12)	0.0314 (11)	0.0390 (13)	−0.0017 (9)	−0.0002 (12)	−0.0019 (12)
C11	0.0449 (14)	0.0307 (12)	0.0489 (15)	−0.0092 (10)	0.0008 (14)	0.0005 (13)
C12	0.0337 (12)	0.0289 (11)	0.0476 (14)	0.0013 (9)	0.0003 (13)	0.0008 (12)
Cl1	0.0305 (3)	0.0333 (3)	0.0362 (3)	0.0070 (2)	−0.0006 (3)	−0.0032 (3)
Cl2	0.0329 (3)	0.0478 (3)	0.0302 (3)	−0.0087 (2)	0.0001 (3)	−0.0025 (3)

### Geometric parameters (Å, º)

**Table:**

Cu1—N1	1.9959 (18)	N7—C12	1.361 (3)
Cu1—N5	2.0002 (18)	N8—C10	1.338 (3)
Cu1—N3	2.0104 (16)	N8—C11	1.361 (3)
Cu1—N7	2.0154 (16)	N8—H8N	0.8600
Cu1—Cl1	2.6195 (5)	C1—H1	0.9300
N1—C1	1.318 (3)	C2—C3	1.348 (3)
N1—C3	1.371 (3)	C2—H2	0.9300
N2—C1	1.334 (3)	C3—H3	0.9300
N2—C2	1.363 (3)	C4—H4	0.9300
N2—H2N	0.8600	C5—C6	1.348 (3)
N3—C4	1.313 (3)	C5—H5	0.9300
N3—C6	1.379 (2)	C6—H6	0.9300
N4—C4	1.341 (3)	C7—H7	0.9300
N4—C5	1.360 (3)	C8—C9	1.347 (4)
N4—H4N	0.8600	C8—H8	0.9300
N5—C7	1.315 (3)	C9—H9	0.9300
N5—C9	1.383 (3)	C10—H10	0.9300
N6—C7	1.333 (3)	C11—C12	1.346 (3)
N6—C8	1.366 (3)	C11—H11	0.9300
N6—H6N	0.8600	C12—H12	0.9300
N7—C10	1.312 (3)		
			
N1—Cu1—N5	174.86 (7)	N1—C1—H1	124.7
N1—Cu1—N3	90.12 (7)	N2—C1—H1	124.7
N5—Cu1—N3	89.71 (7)	C3—C2—N2	105.9 (2)
N1—Cu1—N7	88.92 (8)	C3—C2—H2	127.1
N5—Cu1—N7	89.28 (8)	N2—C2—H2	127.1
N3—Cu1—N7	157.71 (7)	C2—C3—N1	109.6 (2)
N1—Cu1—Cl1	92.27 (6)	C2—C3—H3	125.2
N5—Cu1—Cl1	92.84 (6)	N1—C3—H3	125.2
N3—Cu1—Cl1	98.11 (5)	N3—C4—N4	111.18 (19)
N7—Cu1—Cl1	104.18 (5)	N3—C4—H4	124.4
C1—N1—C3	105.83 (19)	N4—C4—H4	124.4
C1—N1—Cu1	129.29 (16)	C6—C5—N4	106.12 (18)
C3—N1—Cu1	124.85 (15)	C6—C5—H5	126.9
C1—N2—C2	108.0 (2)	N4—C5—H5	126.9
C1—N2—H2N	126.0	C5—C6—N3	109.66 (19)
C2—N2—H2N	126.0	C5—C6—H6	125.2
C4—N3—C6	105.36 (17)	N3—C6—H6	125.2
C4—N3—Cu1	124.45 (14)	N5—C7—N6	110.9 (2)
C6—N3—Cu1	130.19 (14)	N5—C7—H7	124.5
C4—N4—C5	107.68 (18)	N6—C7—H7	124.5
C4—N4—H4N	126.2	C9—C8—N6	106.4 (2)
C5—N4—H4N	126.2	C9—C8—H8	126.8
C7—N5—C9	105.9 (2)	N6—C8—H8	126.8
C7—N5—Cu1	127.25 (17)	C8—C9—N5	109.0 (2)
C9—N5—Cu1	126.77 (15)	C8—C9—H9	125.5
C7—N6—C8	107.9 (2)	N5—C9—H9	125.5
C7—N6—H6N	126.1	N7—C10—N8	110.73 (19)
C8—N6—H6N	126.1	N7—C10—H10	124.6
C10—N7—C12	106.09 (18)	N8—C10—H10	124.6
C10—N7—Cu1	126.20 (14)	C12—C11—N8	106.1 (2)
C12—N7—Cu1	127.67 (14)	C12—C11—H11	126.9
C10—N8—C11	107.46 (18)	N8—C11—H11	126.9
C10—N8—H8N	126.3	C11—C12—N7	109.6 (2)
C11—N8—H8N	126.3	C11—C12—H12	125.2
N1—C1—N2	110.6 (2)	N7—C12—H12	125.2
			
C3—N1—C1—N2	0.4 (3)	C9—N5—C7—N6	−0.3 (3)
Cu1—N1—C1—N2	178.33 (16)	Cu1—N5—C7—N6	−176.65 (17)
C2—N2—C1—N1	−0.4 (3)	C8—N6—C7—N5	0.3 (3)
C1—N2—C2—C3	0.3 (3)	C7—N6—C8—C9	−0.1 (3)
N2—C2—C3—N1	0.0 (3)	N6—C8—C9—N5	−0.1 (3)
C1—N1—C3—C2	−0.2 (3)	C7—N5—C9—C8	0.2 (3)
Cu1—N1—C3—C2	−178.27 (18)	Cu1—N5—C9—C8	176.59 (19)
C6—N3—C4—N4	−0.1 (3)	C12—N7—C10—N8	−0.4 (3)
Cu1—N3—C4—N4	−179.35 (16)	Cu1—N7—C10—N8	177.33 (16)
C5—N4—C4—N3	0.2 (3)	C11—N8—C10—N7	0.3 (3)
C4—N4—C5—C6	−0.3 (3)	C10—N8—C11—C12	−0.1 (3)
N4—C5—C6—N3	0.3 (3)	N8—C11—C12—N7	−0.1 (3)
C4—N3—C6—C5	−0.1 (3)	C10—N7—C12—C11	0.3 (3)
Cu1—N3—C6—C5	179.09 (19)	Cu1—N7—C12—C11	−177.3 (2)

### Hydrogen-bond geometry (Å, º)

**Table:**

*D*—H···*A*	*D*—H	H···*A*	*D*···*A*	*D*—H···*A*
N2—H2*N*···Cl2^i^	0.86	2.40	3.251 (2)	169
N4—H4*N*···Cl2^ii^	0.86	2.52	3.1742 (19)	133
N6—H6*N*···Cl2^iii^	0.86	2.39	3.241 (2)	168
N8—H8*N*···Cl1^iv^	0.86	2.43	3.2523 (19)	159

Symmetry codes: (i) *x*−1/2, −*y*+1/2, *z*−1/2; (ii) *x*−1, *y*, *z*; (iii) *x*−1/2, −*y*+1/2, *z*+1/2; (iv) *x*+1, *y*, *z*.
